# Association between Socioeconomic Status and Healthcare Utilization for Children with Allergic Diseases: Korean National Health and Nutritional Examination Survey (2015–2019)

**DOI:** 10.3390/healthcare11040492

**Published:** 2023-02-08

**Authors:** Jeoungmi Kim, Bomgyeol Kim, Do Hee Kim, Yejin Kim, Vasuki Rajaguru

**Affiliations:** 1 Department of Nursing Science, Kaya University, Gimhae 50830, Republic of Korea; 2 Department of Public Health, Yonsei University, Seoul 03722, Republic of Korea; 3 Department of Healthcare Management, Graduate School of Public Health, Yonsei University, Seoul 03722, Republic of Korea

**Keywords:** key allergic disease, asthma, dermatitis, inequity, disparity, children

## Abstract

This study aimed to investigate the association between socioeconomic status (SES) and healthcare utilization by children with allergic diseases. We determined SES based on parental occupation and household income. A cross-sectional study was conducted using the Korean National Health and Nutritional Examination Survey (KNHANES) between 2015 and 2019 with participants who were under 18 years of age. The presence of allergic conditions was determined by a self-reported survey of parental response and healthcare utilization data (such as inpatient and outpatient visits). Moreover, we categorized SES into four quantiles (Q1–Q4) based on household income per annum. Then, the data were analyzed using chi-square tests and multivariate logistic regression analysis with confidence intervals (CIs) of 95%, and *p* < 0.05 was considered significant. A total of 3250 participants were involved in this study. The percentage of allergic diseases was 67.9% for allergic asthma and 32.1% for atopic dermatitis. It was found that the participants who were over 13 years old had atopic dermatitis and were more likely to visit the hospital than younger children. Additionally, the highest SES group in Q4 demonstrated higher healthcare utilization (OR = 1.58; 95% CI, 1.14–1.76) than other SES groups. Our study reveals that parental socioeconomic characteristics are related to the use of healthcare services for children with allergic disorders in Korea. These results highlight the need for public health actions and research to overcome the SES gap among children with allergic diseases.

## 1. Introduction

Worldwide, especially in low- and middle-income countries, the prevalence of allergic illnesses such as asthma has rapidly increased over recent decades [1]. The impact of the global trends in allergy-related disorders is especially great among vulnerable children and young people [1,2,3]. Although the evidence for these trends in respiratory-related allergic diseases in the context of Korea is debatable, previous research has identified a rising prevalence rate of respiratory-related allergic diseases [3,4]. Although prior research has indicated an increase in both conditions, the evidence for such increases in asthma, allergic rhinitis, and atopic dermatitis in Korea is debatable [3,4,5,6].

Major concerns still persist regarding the age-specific developments in allergic disorders and asthma among the Korean population. Recently, the trend in prevalence has shifted, as the occurrence of most allergic diseases has increased among all the age groups beyond school-age children, and atopic dermatitis prevalence trends vary greatly in specific age groups such as adolescents [6]. This change in trend suggests that the control of moderate allergy diseases that have an impact on quality of life should receive more focus, in addition to the treatment of severe allergic diseases such as asthma [5,6].

Since the 1990s, health disparities have received significant attention in research, particularly the association between socioeconomic status (SES) and the use of healthcare services [7,8,9,10]. Health disparities are primarily caused by numerous social determinants of health and their variations are reported in terms of accessing healthcare services, such as young ages, insurance patterns, lower parental income, low or average household income, and higher incremental costs for allergic diseases [11,12,13]. Despite the rising interest in health inequities, children’s diseases have primarily been explored in depth using various socioeconomic indices, populations, contexts, and inequality measures [13,14]. However, despite the rising interest in health inequities, previous research has demonstrated a very low level of healthcare outcomes for children with respiratory allergic diseases within Europe and the United States [13,14]. Primarily, children’s diseases have been explored in depth using various socioeconomic indices, populations, contexts, and inequality measures [13,14,15].

Numerous studies have demonstrated a relationship between the incidence of allergic diseases, socioeconomic risk factors, and genetic risk factors [3,4,5,6,7]. Moreover, hospital utilization varies between urban and rural areas, and this disparity has frequently been linked to healthcare service access [16]. Reportedly, hospital utilization is higher in urban than rural areas, and hospitals in rural areas typically have less resources than the facilities in urban areas. Furthermore, since the health disparities related to the association between gender and age have already been studied in children [11,12,13,14,15,16,17,18], there is a need for research on the health disparities related to the association between SES and healthcare utilization. This study assumed that healthcare utilization might vary depending on SES categories.

Therefore, this study aimed to examine the association between SES and healthcare utilization along with the five-year incidence of two allergic diseases (i.e., allergic asthma and atopic dermatitis) by using the Korean National Health and Nutrition Examination Survey (KNHANES) from 2015 to 2019.

## 2. Materials and Methods

### 2.1. Data Source and Study Population

The Korea National Health and Nutrition Examination Survey (KNHANES) is a nationwide survey that collects uniform cross-sectional data on the health status, healthcare utilization, and SES of the entire Korean population [6]. Since its inception in 2007, the survey has been carried out annually. KNHANES employs multi-stage cluster sampling and targets non-institutionalized Korean citizens from the household registry, and post-stratification is used to account for cross-sectional study designs. The primary sample units (PSU) were chosen from across Korea. We used the data from KNHANES’s sixth, seventh, and eighth surveys (as the data from the eighth survey are available only till 2019), which asked fundamental questions regarding the participants’ household income and personal traits, including demographics, health, and lifestyle.

KNHANES was authorized by the KDCA Institutional Review Board (2018-01-03-P-A) in 2018, and the research was carried out in accordance with the principles outlined in the Declaration of Helsinki.

### 2.2. Variables and Measurement

#### 2.2.1. The Dependent Variable

The dependent variable of this study was healthcare utilization by children with allergic diseases, and the responses were recorded as either “yes” or “no”. Furthermore, we identified the presence of allergic diseases through the answers given to the following survey question [19]: “Have you ever been diagnosed with asthma/allergic rhinitis/atopic dermatitis by a doctor”?

#### 2.2.2. Independent Variable

The general characteristics included age, gender, parental education and occupation, residence, and health insurance membership. We divided the children into two age groups: (a) below 12 years of age and (b) 13 years of age and above. Moreover, we divided the parental education status of both the mother and the father of a participant into four levels: elementary school, middle school, high school, and university and above. Furthermore, based on their answers, we classified their occupational status as either “yes” (all types of work) or “no” (no work). Additionally, the residential areas were considered as urban (metropolitan city or town) and rural (county or villages) areas.

The individuals’ socioeconomic status was measured in three domains: household income, educational level, and occupational status. Previous studies have utilized household income as a measure of SES rather than individual income. However, this study focused only on income level (quartile) to measure the SES, and it was calculated based on equivalized income (total household income divided by the square root of the number of household members); it was also categorized into four categories: lowest (Q1), middle (Q2), high (Q3), and highest (Q4). The health insurance membership was divided into two categories: National Health Insurance (NHI) (employee benefits) and Medicare (family or self-membership).

### 2.3. Statistical Analysis

The data analysis was conducted in two steps. First, we performed chi-square tests to identify the differences based on the participants’ responses to the presence of allergic diseases, frequency, and percentage for categorical variables or mean (standard deviation) for continuous variables. Then, we employed multivariate logistic regression models to determine the factors associated with healthcare utilization by children with allergic conditions according to healthcare utilization and subgroup analysis for SES; we determined by the odds ratios (ORs) and confidence intervals (CIs) of 95% by adjusting for potential covariates. All the data were analyzed by using SAS version 9.4 (SAS Institute Inc., Cary, NC, USA). A *p*-value < 0.05 was considered statistically significant.

## 3. Results

### 3.1. Study Population and Distribution of Allergic Diseases among Korean Children

**Figure 1:**
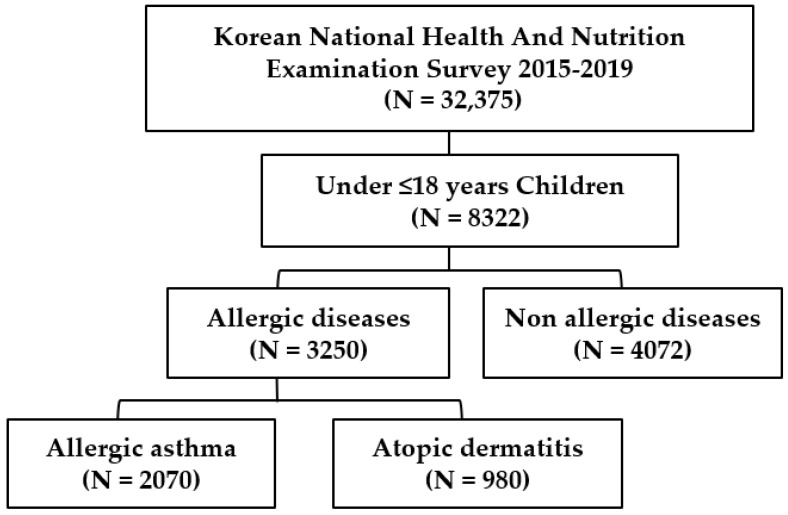
Flow chart for selection of the study population.

**Figure 2:**
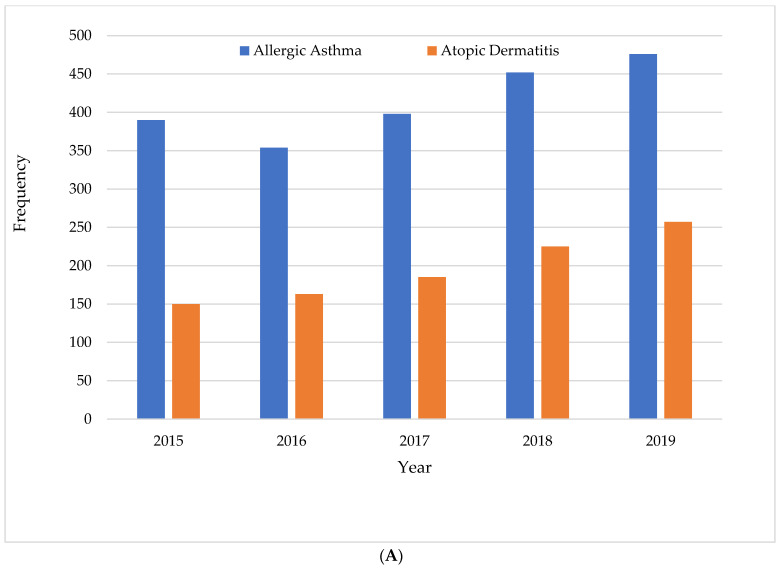

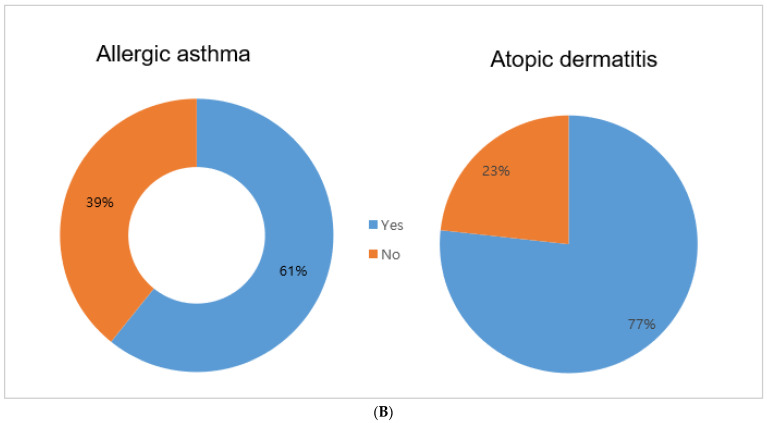
(**A**). Frequency distribution of allergic diseases by year based on the KNHANES data 2015–2019. (**B**). Percentage distribution of allergic asthma and atopic dermatitis.

A total of 32,375 households participated in KNHANES from 2015 to 2019. Out of these, 2070 children with allergic asthma and 980 children with allergic dermatitis were included in this study (Figure 1). The frequency distribution of the allergic diseases during five-year trends and the overall percentage of children with asthma (61%) and atopic dermatitis (77%) are presented in Figure 2A,B.

### 3.2. Characteristics of Study Children with Allergic Diseases

The characteristics of the study population who reported (yes) allergic diseases, i.e., allergic asthma and atopic dermatitis, are presented in Table 1. Most of the participants were girls with allergic asthma (53.6%) and boys with atopic dermatitis (64.1%). Moreover, they were under the age of 12 (87.1%) and over the age of 13 with atopic dermatitis, respectively. Furthermore, in both cases, the fathers (41.2%; 40.8%) and mothers (35.8%; 43.3%) reported having completed high school education. Additionally, most of the parents worked (60.1%; 93.0%) and lived in cities (55.8%; 70.3%). As regards health insurance membership, the children with allergic asthma had NHI (54.3%) and those with atopic dermatitis had Medicare (96.4%).

We found that among children with allergic disorders who utilized healthcare, around 60.8% had asthma and 76.7% had atopic dermatitis. Moreover, the children with allergic asthma had a household income level (or SES) of Q3 (29.5%), while those with atopic dermatitis had an SES of Q4 (21.4%).

### 3.3. Multivariate Logistic Regression Analysis of Factors Associated with Healthcare Utilization for Children with Allergic Diseases

**Figure 3:**
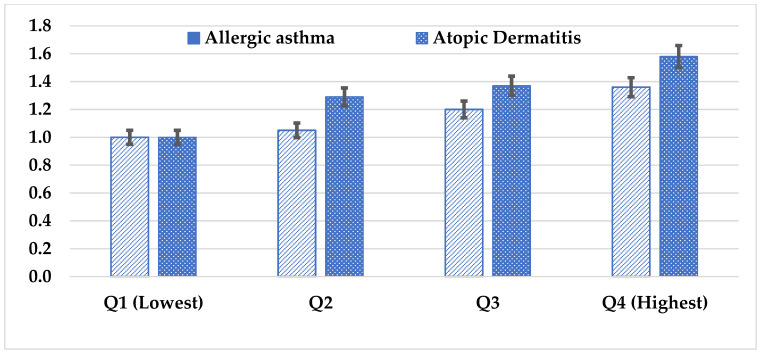
The association between SES and healthcare utilization for children with allergic conditions. Adjusted odd ratio with age, gender, parental education and occupation, residence, and health insurance.

The results of the multivariate logistic regression analysis were obtained among the children who had healthcare utilization and associated factors. The associations between general characteristics and healthcare utilization for children with allergic diseases are given in Table 2. Concerning the likelihood of healthcare utilization, girls exhibited higher rates of both allergic asthma (OR = 1.36, 95% CI: 1.08–1.71) and atopic dermatitis (OR = 1.38, 95% CI: 1.24–1.54) compared to the boys. Moreover, when comparing children of different age groups, it was found that those over the age of 13 with atopic dermatitis had a higher (OR = 1.37, 95% CI) likelihood of utilizing healthcare than other age groups, which was statistically significant at the *p*-value of 0.001. On the other hand, it was found that the children with allergic diseases who lived in rural areas had a lower likelihood of using healthcare, and patients with NHI were more likely to utilize healthcare than those with a self-health insurance membership. In atopic dermatitis, Medicare membership (OR = 1.11, 95% CI) was related to the higher utilization of healthcare than NHI membership. The result of the subgroup analysis of SES is presented in Figure 3. Overall, the highest income group’s Q4 healthcare consumption was higher than that of the other SES groups for both allergic asthma (OR = 1.36, 95% CI: 1.08–1.71) and atopic dermatitis (OR = 1.58, 95% CI: 1.41–1.76), which was statistically significant at the *p*-value of 0.001.

## 4. Discussion

This study aimed to examine the association between the SES and the healthcare utilization of children with allergic diseases using the KNHANES data from 2015–2019. Social status and the incidence of allergic diseases and atopy were considered. We found a higher prevalence of allergic asthma and atopic dermatitis, as primary respiratory-based diseases, than allergic rhinitis. Therefore, we included two allergic diseases: allergic asthma and atopic dermatitis. According to the five-year trends in allergic disease incidence in Korean children, allergic asthma and atopic dermatitis increased gradually between 2015 and 2019. Over the past few decades, the occurrence of allergic diseases has significantly increased, especially in developing nations [1]. The incidence of allergic diseases over a 12-month period, the prevalence of diagnosis, and the prevalence of treatment increased by more than 10–15% as compared to the national research conducted in 2000 and 2006 [19,20].

Several important findings are demonstrated in this study. Atopic dermatitis affected 32.1% and allergic asthma affected 67.9% of the 3250 Korean children in this population-based sample. Similar research has found that 46.3–50.4% of Asian infants suffer from allergic disorders [2,4,14,16,20,21].

Our findings showed the most of the girls had allergic asthma and boys had atopic dermatitis. Generally, allergic diseases were generally more likely to be associated with atopic dermatitis in various time periods, due to the hormonal and immunological changes among adolescents compared to younger children [22,23,24]. In this study, the parental education level and monthly household income were associated with healthcare utilization. Previous studies have reported similar results, i.e., allergic asthma is related to the parental education level and household income [25,26].

The healthcare utilization findings revealed that most of the participants residing in the urban area visited healthcare settings, including as an inpatient and outpatient, more often than those in rural areas. These results are supported by findings that the inequalities in hospital utilization between urban and rural communities are frequently attributed to the justice gap and access to healthcare [16]. Hospital utilization is higher in urban than rural locations, and hospitals in rural areas typically have less resources than facilities in urban areas [11,16,24]. Therefore, necessary steps should be taken regarding healthcare access among children with allergic diseases to improve their healthcare access.

The study results revealed that the highest SES (Q4) group showed higher healthcare utilization than the lowest SES group (Q1). This is because individuals with lower incomes commonly struggle to afford medical costs. Overall, it was found that there were many significant variables associated with the use of healthcare utilization in the high-income group. As a result, the socioeconomic status of an individual, which affects their burden of spending on such care services, was identified as a specific factor contributing to the poor utilization of healthcare services. Numerous studies have reported that SES is associated with healthcare utilization according to parental education and household income [9,12,17,18,24,27]. Another study reported that the burden of medical expenses for preschool children is lower than in other nations due to universal medical insurance and regional government subsidies for children’s medical expenses [28]. Further research is required to validate our findings and investigate possible interpretations.

Our research demonstrates that despite the important role that public health policies play in ensuring that children with allergic disorders have free and equitable access to medical care, health status equity is not always achieved. Thus, similar findings will encourage governments to address social determinants as an important part of healthcare access in order to reduce health disparities.

### Strengths and Limitations

We believe that this is the first study that has focused on the SES and healthcare utilization of children with allergic diseases. These national-based data comprise the entire population of children under 19 years old, across a five-year period, offering significant potential to identify more children with low-prevalence allergic diseases.

There are a few limitations in this study. This study focused on a cross-sectional design, which makes it impossible to determine the timing of the association between atopic dermatitis and allergic asthma. However, the majority of published articles assessing the proportion of allergy diseases carry a possibility of possessing this restriction. We did not include allergic rhinitis, since it is considered a seasonal condition; in addition, due to differences in exposure to healthcare utilization, the attributable risk of allergic diseases may differ between nations. Further study could be conducted on this aspect for a better outcome. In addition, this study was based on a self-administered survey with some level of subjectivity, which may have created bias. For instance, adolescents tend to have atopic dermatitis, which can distort data on allergic diseases in children below the age of 13 years. Our findings may not apply to other Asian nations; thus, they should be interpreted in the context of Korea only. We used open data from KNHANES and additional information related to other health outcomes was not included. Healthcare utilization and SES were considered according to the parents’ survey responses. Therefore, it is anticipated that self-reported household income or occupation may create healthcare utilization concerns, which requires further research.

## 5. Conclusions

In the present study, we found an association between healthcare utilization and the highest SES group. Our study suggests that approximately half (87.1% and 64.5%) of Korean children have allergic diseases that are attributable to allergic asthma and atopic dermatitis. We also found that the group over 13 years of age was more vulnerable to atopic dermatitis and more likely to utilize healthcare services than younger children. Therefore, there is a need for further research on the factors related to SES and healthcare utilization according to the household income level and the specific age group of adolescents using the National Health Insurance Service data. Overall, the study findings suggest that, in order to reduce the barriers associated with family socioeconomic background and the disparities in healthcare utilization, policymakers should devise consistent health policies regarding the use of healthcare facilities, the quality of health services, and health insurance. Future policies that aim to address healthcare utilization associated with allergic diseases and SES disparities must consider various household income patterns according to SES and age group. Specifically, a needs-based intervention or policy strategy may be necessary to target lower-socioeconomic-status (SES) households with young children.

## Figures and Tables

**Table 1:** General characteristics and distribution among children with allergic diseases (*n* = 3050).

Variables		Allergic Asthma		Atopic Dermatitis		
		Yes		Yes		
		N	%	N	%	*p*
		2070	67.9	980	32.1	
Gender	Boys	961	46.4	628	64.1	<0.001
	Girls	1109	53.6	352	35.9	
Age (Years)	Upto 12	1802	87.1	348	35.5	<0.001
	Over 13	268	12.9	632	64.5	
Father’s education	Elementary school	110	5.3	70	7.1	
	Middle school	398	19.2	200	20.4	0.352
	High school	852	41.2	400	40.8	
	University and above	710	34.3	310	31.6	
Mother’s education	Elementary school	140	6.8	51	5.2	0.412
	Middle school	608	29.4	325	33.2	
	High school	742	35.8	424	43.3	
	University and above	580	28.0	180	18.4	
Occupation	Yes	1245	60.1	911	93.0	<0.001
	No	825	39.9	69	7.0	
Residence	Urban	1156	55.8	689	70.3	<0.001
	Rural	914	44.2	291	29.7	
Health insurance	NHI	1125	54.3	945	96.4	<0.001
	Medicare	945	45.7	35	3.6	
Household income (SES)	Q1 (Lowest)	501	24.2	139	14.2	<0.001
	Q2	579	28.0	473	48.3	
	Q3	610	29.5	158	16.1	
	Q4 (Highest)	380	18.4	210	21.4	
Healthcare utilization	Yes	1258	60.8	752	76.7	<0.001
	No	812	39.2	228	23.3	

**Table 2:** Multivariate logistic regression analysis for factors associated with healthcare utilization in children with allergic diseases (*n* = 2010).

Variables	Healthcare Utilization (Yes)					
	Allergic Asthma (*n* = 1258)			Atopic Dermatitis (*n* = 752)		
	aOR *	95% CI	*p*	aOR *	95% CI	*p*
Gender						
Boys	Ref			Ref		
Girls	1.36	1.08–1.71	0.018	1.38	1.24–1.54	<0.001
Age (Years)						
Upto 12	Ref			Ref		
Over 13	0.55	0.32–0.98	<0.001	1.37	1.08–1.53	<0.001
Residence						
Urban	Ref			Ref		
Rural	0.98	0.95–1.02	0.335	0.74	0.41–1.33	0.268
Household Income						
Q1 (Lowest)	Ref			Ref		
Q2	1.05	1.00–1.10	0.003	1.29	1.14–1.46	<0.0001
Q3	1.2	1.04–1.38	0.021	1.37	0.58–3.23	0.152
Q4 (Highest)	1.36	1.08–1.71	<0.001	1.58	1.41–1.76	<0.001
Health Insurance						
NHI	1.00			1.00		
Medicare	0.88	0.84–0.93	<0.001	1.11	1.01–1.13	<0.001

aOR = adjusted odds ratio, CI = confidence interval; NHI = National Health Insurance. * Adjusted for age, gender, parental education and occupation, residence, and health insurance.

## Data Availability

The data presented in this study are available on request from the corresponding author.
